# Hidden Hunger: A Pellagra Case Report

**DOI:** 10.7759/cureus.14682

**Published:** 2021-04-25

**Authors:** Hugo Pinheiro, Margarida Matos Bela, Ana Filipa Leal, Luís Nogueira, Mari Mesquita

**Affiliations:** 1 Internal Medicine, Tâmega e Sousa Hospital Center, Penafiel, PRT

**Keywords:** pellagra, niacin

## Abstract

Pellagra is a deadly nutritional disease caused by niacin deficiency. Although practically eradicated in developed countries, it still affects vulnerable populations. The diagnosis is based on the presence of characteristic dermatitis in sun-exposed areas, diarrhea, and dementia. We report the case of a woman with a clinical picture of hyperpigmentation and hyperkeratinization in exposed areas of the skin, watery diarrhea, and progressive disorientation with disorganized speech. The anamnesis revealed a poor diet regimen composed almost exclusively of cassava root meals. Alternative diagnosis was excluded and nicotinamide supplementation was introduced with progressive resolution of symptoms until complete recovery. This case report highlights the need to maintain a high index of suspicion in the presence of characteristic symptoms for timely diagnosis of this deadly condition with a simple but dramatic curative treatment.

## Introduction

Pellagra is a nutritional disease caused by deficiency of niacin (also known as nicotinic acid or vitamin B3) and/or of its precursor tryptophan [1]. Although practically eradicated in developed countries, sporadic cases of pellagra continue to appear, particularly in vulnerable populations [1]. The diagnosis is clinical and is based on the presence of characteristic dermatitis in sun-exposed areas, diarrhea, and dementia, three of the four D’s of pellagra [2].

Niacin is essential for normal cellular metabolism as a precursor to nicotinamide adenine dinucleotide (NAD) and nicotinamide adenine dinucleotide phosphate (NADP) [1,3]. Therefore, tissues with high turn-over rates, such as the skin and the bowel, or with high energy consumption, such as the brain, are the most affected by niacin deficiency [3].

## Case presentation

**Figure 1:**
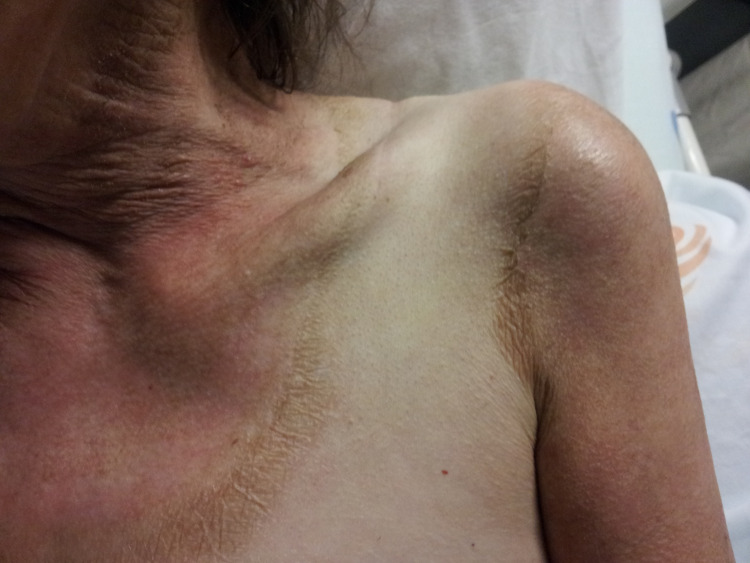
Characteristic ring of erythema and hyperpigmentation extending around the neck onto the chest in pellagra (known as Casal’s necklace).

A 69-year-old woman with alcohol use disorder (estimated consumption of 50-60 g/day) was brought to the emergency department with progressive disorientation, and disorganized speech until abulia over the previous two months. Fourteen days before the emergency episode the patient developed watery diarrhea. The anamnesis revealed that, on the last three to four months, patient’s diet consisted almost exclusively of cassava root meals. On admission the patient was dehydrated, pale, lethargic, disoriented, and bradypsychic. Blood pressure was 109/66 mmHg, with a regular pulse of 77 beats/min. Axillary temperature was 36.6 ºC and peripheral capillary oxygen saturation was 97%. Body mass index was 16.26 kg/m^2^. The patient displayed symmetrical scaly rash with hyperpigmentation and hyperkeratinization in exposed areas of the skin (Figure 1).

The initial bloodwork revealed hypoproliferative macrocytic anemia (hemoglobin 7.2 g/dL, mean corpuscular volume 99 fL, reticulocyte index 0.29), 3400 leukocytes/µL, 114,000 platelets/µL, C-reactive protein of 32.6 mg/L (reference <7.5 mg/L), hypoalbuminemia of 1.7 g/dL (reference 2.5-4.8 g/dL), and hypoproteinemia of 4.1 g/dL (reference 6.1-7.9 g/dL) with mildly elevated bilirubin of 1.20 mg/dL (reference <1.00 mg/dL) and normal renal function (creatinine 0.4 mg/dL, no proteinuria or casts). Severe hypokalemia was also documented (1.7 mmol/L, reference 3.5-5.1 mmol/L) without other ion alterations. Coagulation was normal. Ammonia levels were within normal range (33 µmol/L, reference 26-50 µmol/L) and urine was negative for drugs. The electrocardiogram and chest radiograph were normal, and the brain computed tomography scan showed mild ischemic leukoencephalopathy, small deep lacunar infarcts, and mild medial temporal atrophy.

The patient was admitted to the medical ward for further work-up. The investigation revealed low levels of folate (2.1 ng/mL; reference 3.1-19.1 ng/mL), normal levels of vitamin B12 (251 pg/mL; reference 180-914 pg/mL), low serum iron (33 µg/dL; reference 49-151 µg/dL), low transferrin levels (91 mg/dL; reference 192-382 mg/dL), and high ferritin (1046 ng/mL; reference 11-307 ng/mL). Chronic infections (such as tuberculosis, hepatitis, HIV, Lyme’s disease, and Whipple’s disease) and cancer were ruled out. At this point, based on the history, physical examination, and laboratory findings, the diagnosis of pellagra was made.

We started supplementation with nicotinamide (300 mg/day) and folate (5 mg) associated with a high-protein diet fed through a nasogastric tube. The diarrhea resolved, and the neurological state improved gradually, allowing nasogastric tube removal and feeding by mouth. At discharge, the patient was alert and oriented to time and place and the speech was poor but clear and fluent. At home, the patient was fed a high-protein diet, multivitamin supplements, and maintained a daily dose of 300 mg of nicotinamide. On re-evaluation, the patient continued to gain weight and functionality until full recovery after one year of treatment with complete resolution of the skin lesions.

## Discussion

Pellagra skin lesions are characteristic and pathognomonic [3]. Initially, the lesions present as a symmetrical and bilateral erythematous non-pruritic rash, which is well defined in areas of sun exposure, friction, or pressure, changing into a cinnamon-brown color [2]. Occasionally, vesicles and blisters can be found, particularly in acute pellagra and re-exposure to sunlight [2]. A characteristic feature of the disease is Casal’s necklace, which is a ring of erythema and hyperpigmentation extending around the neck onto the chest [2]. Inflammation and atrophy of the entire gastrointestinal tract results in anorexia, nausea, and watery diarrhea, which ultimately leads to perpetuation of the vicious cycle by decreasing nutrient absorption [2-4]. Neurological manifestations are variable and usually present in the advanced stages of the disease [3]. Insomnia, irritability, and depression may be found and progress to encephalopathy characterized by confusion, memory loss, and psychosis [2]. In more advanced stages, delirium ensues, followed by stupor, coma, and finally death (the last of pellagra’s four D´s) [2].

The diagnosis is further supported by the presence of anemia, hypoproteinemia, hypercalcemia, hypokalemia, and hypophosphatemia [2]. In susceptible populations such as those with chronic alcoholism, liver cirrhosis, anorexia nervosa, wasting conditions (carcinoid tumors), or under some medications, the diagnosis may be missed using the classic dermatitis, diarrhea, and dementia [2,4,5]. Excessive and chronic alcohol intake is associated with increased malnutrition and can induce pellagra both by reducing niacin absorption and impairment of tryptophan conversion to niacin [6]. There are no tests available to definitively diagnose pellagra, but serum levels of niacin, tryptophan, NAD, and NADP, as well as the urinary metabolites N′-methyl-nicotinamide and N′-methyl-2-pyridone-5 carboxamide can be measured to support the diagnosis [2,3]. However, testing for these substances is available only in expert centers.

The administration of niacin has a dramatic curative impact on pellagra. Large amounts of niacin should be provided in the form of nicotinamide, which does not produce the side effects encountered when nicotinic acid is administered [2]. The daily recommended intake is 300 mg of nicotinamide in divided doses, and treatment should continue for three to four weeks [4]. Acute inflammation of the tongue and mouth, as well as diarrhea, subside in a few days. The dementia and dermatitis usually improve significantly within the first week of therapy. In chronic cases, a longer recovery period is required, but appetite and general physical health improve rapidly. It is also recommended to administer a vitamin B complex preparation or a yeast product since patients with pellagra very often have a deficiency of other B vitamin compounds [7].

## Conclusions

Although practically eradicated in developed countries, sporadic cases of pellagra still exist. The diagnosis is based on the presence of characteristic three D’s of pellagra, dermatitis in sun-exposed areas, diarrhea, and dementia. The maintenance of a high index of suspicion is advised in the presence of any of the symptoms, particularly in vulnerable populations to ensure the diagnosis and timely treatment of this potentially lethal condition.
